# Research utilization among children's mental health providers

**DOI:** 10.1186/1748-5908-3-19

**Published:** 2008-04-09

**Authors:** Melanie A Barwick, Katherine M Boydell, Elaine Stasiulis, H Bruce Ferguson, Karen Blase, Dean Fixsen

**Affiliations:** 1 Community Health Systems Resource Group, The Hospital for Sick Children, Toronto ON, Canada; 2 University of Toronto, Toronto ON, Canada; 3 Louis de la Parte Florida Mental Health Institute, National Implementation Research Network, Florida, USA; 4 University of South Florida, Florida, USA

## Abstract

**Background:**

Children with emotional and behavioural disorders should be able to count on receiving care that meets their needs and is based on the best scientific evidence available, however, many do not receive these services. Implementation of evidence-based practice (EBP) relies, in part, on the research utilization practices of mental health care providers. This study reports on a survey of research utilization practices among 80 children's mental health (CMH) service provider organizations in Ontario, Canada.

**Methods:**

A web-based survey was distributed to 80 CMH service provider organizations, to which 51 executive directors and 483 children's mental health practitioners responded. Research utilization was assessed using questions with Likert-type responses based on the Canadian Health Services Research Foundation's Four-A's approach: access, assess, adapt, apply.

**Results:**

There was general agreement among executive directors and practitioners regarding the capacity of their organizations to use – access, assess, adapt, and apply – research evidence. Overall, both groups rated their organizations as using research information 'somewhat well.' The low response rate to the practitioner survey should be noted.

**Conclusion:**

These findings provide a useful benchmark from which changes in reported research utilization in the Ontario CMH sector can be tracked over time, as a function of EBP training and implementation initiatives, for instance. The need to improve access to research evidence should be addressed because it relates to the eventual implementation and uptake of evidence-based practices. Communities of practice are recommended as a strategy that would enable practitioners to build capacity in their adaptation and application of research evidence.

## Background

Approximately one in five children have a diagnosable mental disorder and one in ten youths have a serious emotional or behavioural disorder that is severe enough to cause substantial impairment in functioning at school, at home, or in the community [1]. Approximately 75 percent of children with emotional and behavioural disorders do not receive mental health services [2]. Those that do receive care often receive treatments and interventions that are not based on evidence of efficacy or effectiveness [3]. This context creates a pressing agenda for the implementation of evidence-based practices in children's mental health service delivery.

This project set out to explore research utilization barriers and facilitators among executive leaders and practitioners in children's mental health (CHM) organizations in Ontario. Specifically, we were interested in learning how CMH organizations and practitioners, access, assess, adapt, and apply evidence-based research knowledge into their every day care for children and youth.

The term 'evidence-based practice' (EBP) refers to a body of scientific knowledge about a range of service practices (*e.g.*, referral, assessment, case management, therapies, or support service) [4]. The implementation of evidence-based practices in Ontario's children's mental health system requires a dual effort: providing the financial resources and public agenda that ensures children and youth receive services on the basis of need not availability, and ensure that the services provided are of the highest quality and most scientifically sound. While the government must address the financial aspects of this agenda, the children's mental health sector is challenged to move forward on the accountability and quality front, and incorporate EBPs into usual care. However, it is not sufficient to build the Cadillac of implementation strategies in the absence of understanding what is needed to prepare organizations and practitioners in the field to receive and implement this new knowledge. An examination of the research utilization practices of CMH service providers within a system of care can elucidate common research utilization practices and related barriers experienced by CMH practitioners and leaders, and thereby contribute to the development of effective and efficient strategies to support successful EBP implementation.

Research utilization is defined here as the use of research to guide clinical practice [5]. Little is known about the characteristics of research utilization in mental health, and even less in child and youth mental health. The practitioner and executive director research utilization practices reported here are but one aspect of a larger survey [6] that examined organizational readiness for change among CMH organizations and practitioners.

Empirical studies on research utilization by practitioners in mental health are few [7]. As a consequence, theoretical and empirical work is needed in order to better understand the facilitators of research use by mental health practitioners. More broadly, the area of implementation science has focused on the study of methods to promote the uptake of research findings for the purpose of improving quality health care [8]. A review of the literature suggests that providing mental health services that are backed by evidence of effectiveness is increasingly important [9-15]. Indeed, four of the eight goals on the agenda of the Surgeon General's Conference on Children's Mental Health [15] pertain to increased implementation of scientifically proven prevention and treatment services. In an example of how this direction can be operationalized, the report of the National Advisory Mental Health Council's Workgroup on Child and Adolescent Mental Health Intervention, Development and Deployment [14] encouraged the development of Treatments and Services Practice Networks to examine the transfer and quality improvement strategies for implementing EBPs.

The evidence is fairly strong in claiming that the passive transfer or dissemination of clinical practice guidelines or other evidence, in isolation of other strategies is insufficient to attain practice change [16,17]. There is much support for the claim that simply disseminating clinical practice guidelines is ineffective for influencing the behaviour of practitioners. There is some consensus that the implementation of EBPs is a shared responsibility of organized systems of care (state, province), professional and consumer organizations, and individual practitioners [16,18-22], and that the successful implementation of evidence into practice requires attention to multiple levels and processes: the practitioner, the organizational context, the system of care context, the nature of the evidence, and the method(s) of transfer and implementation [17,22-28]. Improved understanding of the attitudes service providers hold has been identified by some investigators as necessary to effectively tailor implementation efforts [11,18,28,28-31]. Simply providing practitioners with access to the knowledge base, while important, is not sufficient for the success of EBP knowledge and subsequent implementation [18,32]. The implementation of EBPs requires that practitioners ask searching questions about their practice and service outcomes, incorporating active strategies in professional development and reflective practice [18,33-38]. There is recognition of the importance of organizational and system level leadership in support of implementation efforts [10,18,38], and that factors that can affect success of the innovation be identified at the planning and design stage [39,40]. Lastly, there is a need to study and reconcile the importance of treatment fidelity with the reality that some degree of adaptation of an EBP is typically required in consideration of the environmental context [39,41,42].

## Methods

### Participants

Child and youth mental health services provider organizations that were members of Children's Mental Health Ontario (CMHO) comprised the focus of this study, which was commissioned and funded by CMHO. Children's Mental Health Ontario (CMHO) is an accreditation and advocacy organization that works to improve the mental health and well-being of children and youth and their families. They represent and support the providers of child and youth mental health treatment services throughout Ontario, and had a core membership of 80 community-based children's mental health centres that serve some 150,000 children and their families annually at the time this research was conducted. In Ontario, children's mental health services are delivered by over 100 dedicated children's mental health agencies, over 200 other social service agencies, and two facilities directly operated by the government. Most children's mental health services are delivered through non-profit organizations that operate as children's mental health agencies as well as through inpatient and outpatient programs funded by the Ministry of Health and Long-Term Care (MOHLTC). While some community agencies were explicitly established to offer mental health services, others are offered within the context of a varied menu of community services and may have other primary service foci (*e.g.*, Children's Aid Society, housing agencies, family court services, etc). The additional services provided in general and psychiatric hospital settings through MOHLTC further contribute to the diversity of contexts in which mental health services to children are delivered.

### Measures

Two equivalent survey forms (executive director, practitioner) were developed using a web-based survey application (surveymonkey.com) and distributed electronically. The survey included a four-point Likert scale (not well, somewhat well, well, very well) for each of the four constructs to determine how well the respondents perceived they were doing relative to that area (*e.g.*, How well is your organization able to ACCESS (find and obtain) research-based knowledge?), in addition to questions designed to address barriers to the four constructs (*i.e.*, What barriers are faced by your organization in ACCESSING research-based knowledge? (mark all that apply: time (for seeking & reviewing material; level of difficulty or research material; too much information (overwhelming); lack of resources (money); lack of resources (staff); lack of resources (web access); no barriers; other (response requested).

#### Procedure

A web-based strategy was preferred over traditional paper mailing because of efficiencies in data collection and cost reduction [43]. A letter describing the purpose of the study and providing URL links to the surveys was sent by electronic mail to executive directors in 80 community-based CMH service provider organizations, with a request that they complete the executive director survey and circulate the practitioner survey within their organization. A text version of the practitioner survey was included as an attachment to the email communication, to be circulated to those for whom the web version presented a barrier. To increase the response rate, the letter and its attachments were re-circulated to executive directors on a weekly basis, beginning at the onset of the survey (21 June 2004) until the last week (19 July 2004).

Non-identifiable survey responses were automatically collected in a secure database on the SurveyMonkey™ server and exported to SPSS at the close of the survey. Consent to use the data was implied through completion of the survey. The research protocol was approved by the Research Ethics Board of the Hospital for Sick Children.

## Results

### Response rate

Fifty-eight (72.5%) of the 80 executive directors solicited responded to the survey. Executive directors were asked for a total of clinical staff (part time and full time), so that we could determine a response rate for the practitioner sample. Note that only 58.5% of executive directors indicated they had circulated the practitioner survey to their staff despite numerous reminders. A total of 483 practitioners responded out of an estimated 3,951 staff across the 80 organizations (12.2%). While this low response rate is a limitation from a sampling bias perspective, the data are still valuable insofar as they address the research utilization needs of a large group of CMH practitioners.

Of the 483 practitioners, 405 completed the web version of the practitioner's survey, and a further 78 completed the Word version and returned the survey by fax or mail. Practitioners were well distributed across the province.

### Respondent characteristics

Executive directors reported largely clinical rather than management backgrounds, particularly in social work (48.3%) and psychology (19%). Only 3.4% came from social services, 5.2% from child and youth care, and 5.2% from public health administration. Practitioners' backgrounds were mainly in social work (41.6%) and child and youth care (22.2%), with only 9.8% having backgrounds in psychology. Three quarters of executive directors (77.8%) reported greater than 16 years of clinical experience in the children's mental health field, compared to only one-third of practitioners (36.3%). Seventy percent of executive directors reported over 16 years of management experience. Two-thirds (65.7%) of practitioners identified themselves as frontline staff, 18.3% as 'clinical managers not providing service,' and 16% as 'clinical managers also providing service.'

### Organizational characteristics

Service provider organizations were situated in urban (77.8%), rural (61.1%), and suburban (33.3%) service areas, with some having sites located across these types of regions. Regional representation was assessed as a function of the distribution of the 80 organizations across the province's nine geographic regions. Eight of nine regions showed greater than 50% regional executive director participation, while one region had a representative response rate of 12.5% which may have been due to its having a higher proportion of bilingual and Francophone serving organizations. Fewer than 10% had annual budgets of under $1 million or over $11 million, with 50% reporting annual budgets in the $1 to 5 million range, and 35.2% in the $6 to 10 million range.

#### Internet access

In this day and age, access to the evidence base is highly dependent upon access to the internet and electronic databases. Respondents were asked to indicate the likelihood that their organization would use internet based resources (unable/no access, very unlikely, unlikely, likely, very likely). The majority of executive directors (88.5%) and practitioners (83.3%) reported use of internet resources as 'likely' or 'very likely'. All executive directors were connected to the web from their offices, while 92% of practitioners connected at their desk and 7.6% connected from another location in their workplace. Connection to the internet, and indeed, to system-wide intranets is quite problematic for service providers in rural and remote areas of the province. A lower level of computer use and sophistication has been documented, with internet access in rural areas tending to be slower and less convenient, due to factors such as fewer telephone lines and the continued existence of party lines [44,45]

### Academic access

For many organizations, access to the evidence base is realized because individual staff are connected or affiliated with colleges or universities. These linkages come about through interpersonal interactions with others in these environments, and as a function of access to college or university library systems through academic affiliation. Survey results demonstrated that 67% of practitioners and 77% of executive directors are affiliated with a college or university, either through their role in student supervision, a faculty appointment, or through involvement in continuing education. However, fewer than 40% of participating organizations have membership access, electronic or otherwise, to a university or college library, and this is very likely an important access barrier that could be addressed.

### Organizational capacity for research utilization: access, assess, adapt, adopt

The 'four A's' concept – access, assess, apply, and adapt – was proposed by the Canadian Health Services Research Foundation [46] to capture the essential elements of an organization's capacity for research utilization: 'many organizations would like to make better use of research but aren't sure where to start. Others feel they are doing well, but would also like to know if there are areas in which they could improve' [47]. Survey questions explored whether organizations can: find the research evidence they need (access); assess whether the research is reliable, of high quality, relevant, and applicable (assess); adapt the information to suit its needs, client population, and environment (adapt); and implement and adopt the research information in their context (apply). This framework also was used in an earlier research study with multiple stakeholders and sectors involved in Ontario's children's mental health system [32]. The CHSRF concept of 'adapt' is defined somewhat differently than our application. In the CHSRF self-assessment, 'adapt' refers to the organization's ability to present its own generated research evidence to decision-makers in a useful format that synthesizes recommendations, conclusions, and key issues. Because most children's mental health service providers do not produce their own research, our use of the 'adapt' concept pertains to the organization's ability to use research knowledge to suit its context.

**Figure 1:**
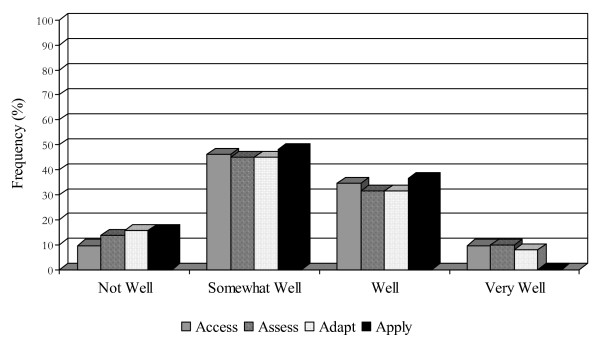
Research utilization among executive directors.

**Figure 2:**
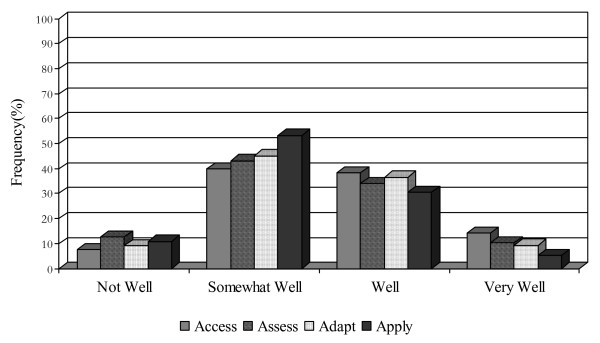
Research utilization practices among practitioners.

An organization's ability to find and obtain research evidence is central to its capacity to evolve with new innovations. Fewer than half of executive directors and practitioners (46.2%, 39.8%) perceived their organization as doing 'somewhat well' in this regard (see Figures 1 and 2). Executive directors and practitioners regarded time (84%, 81%), money (both 51%), and staff (52%, 65%) as the most common barriers to access. Other barriers included lack of access to university libraries, due to distance, lack of financial assistance to obtain materials, and lack of staff to assist in the research and location of materials.

Also noted was poor access to the French-language research literature. Given that there are an estimated half a million Francophones [48] living in Ontario, of which 137,870 are under 25 years of age (comprising 3.8% of Ontario's young people as a whole), access to the evidence base regarding services for this population is important [49]. Practitioners expressed concern regarding barriers at the organizational and/or systemic levels, identifying priority setting, organizational commitment, and time spent on mandated clinical screening and outcome management activities. There was recognition of the need to organize and prioritize accessing research based knowledge and evidence-based practices, and the need to incorporate this knowledge in the supervision experience; something that might be achieved by training clinical supervisors. Many see the biggest barrier as being systemic, referring specifically to the lack of an organizational commitment that allows and fosters front line staff to have time to collect, review, and implement relevant material:

'I believe, in theory, there is a commitment to allow staff the time to read, collect, and research materials. However, in practice, this commitment falls short when faced with prioritizing other demands, such as long wait lists, calls from community members, and the day-to-day clinical momentum of the families with whom we work.'

Still others expressed concern regarding the availability and/or quality of the research evidence, and the bias toward lab-initiated research evidence as opposed to field-level effectiveness research and evaluation of 'promising' practices.

Some practitioners identified the formats and venues for access to the research base as a central barrier, noting that information is better transmitted through training and similar formats but that the availability of finances become the roadblock. In addition, time and resources need to be earmarked so that practitioners can take part in these linkage and exchange opportunities. Often, conferences and other avenues of acquiring specialized knowledge and/or training are not readily available to clinicians, and they can also be a strained on limited financial resources. Lastly, practitioners may not always be aware of where they require additional training.

Attitudes regarding the role research evidence plays in informing clinical practice were also of concern. There was both a sense of inadequacy relative to research utilization, and a sense that 'much of the clinical work can effectively be carried out without research-based knowledge.'

Executive directors and practitioners identified a number of sources used to access research information, showing a similar pattern for the most frequently endorsed formats, such as conferences (89.7%, 82%), journals (81%, 72%), newsletters (60.3%, 45.5%), contact with other organizations (55.2%, 41%), advisory committees (51.7%, 51.9%), and self-directed activities of motivated staff (48.3%, 46.4%). There was variation in the nature of these activities across organizations; with some organizations employing a 'best practices committee to discuss and examine materials,' while others faltering due to a perceived absence of leadership: 'we don't have leadership in this area or designated person to take responsibility so each to his own, every office to its own.'

The majority of executive directors and practitioners (45.1%, 53.2%) rated their organization's capacity to assess the reliability and quality of research as 'somewhat well' (see Figures 1 and 2). Methods used to assess the quality of research evidence included relying on the credibility of the source or the author affiliation (81%, 52.2%), relying on the credibility of the sourcing organization (72.4%, 47%), and staff member research knowledge (67.2%, 55.3%). Approximately half of executive directors would seek consultation (51.7%), contact an expert (48.3%), or assess based on an individual authors' credibility (48.3%). None of the executive directors and only a handful (4%) of the practitioners would consider foregoing some assessment of research reliability.

Only 45.1% of executive directors and practitioners perceived their organization had the capability to modify research information to meet the needs of clients and/or programs 'somewhat well' (see Figures 1 and 2). Fewer than 10% of both groups felt they are doing this 'very well,' suggesting there is room for improvement in this regard and that perhaps specific training on fidelity and 'reinvention' of EBPs is required.

Barriers to the adaptation of research evidence included a lack of research summaries, brief reports, and identified main messages. When asked to identify other barriers to research adaptation, practitioners overwhelmingly identified limited resources, particularly staff time, as an important barrier:

'I work in a residential treatment facility and the environment is always fast paced and (we are) often experiencing various crises. What information we do receive is lost quickly because no time is allotted for staff (to) sit down, free of the front line obligations, to consider the material, review how we can implement what we think would benefit us, etc. Simply put, getting the information is one thing...freeing up the front line staff, the primary ones who will be implementing it on a daily basis to develop ways of adapting it, is the piece that is often overlooked. Without that second piece, the first is negated.

A second very common barrier was staff resistance to the research evidence, as captured in the following:

'(There is) resistance by some staff if (the) evidence-based practice does not fit within their clinical orientation and training. Some staff perceive inclusion of evidence-based practices as too 'top-down' and prescriptive.'

The notion of fidelity and the concern that evidence-based practices would lose their validity and reliability if modified was also identified as a barrier. There was a belief that evidence-based practices are not intended to be modified, and if they are, they will deter from the empirical findings

'Sometimes the model that is being used requires it to be applied in its exact form to ensure fidelity of the program and the expected results. An example of this is the Incredible Years Program.'

Both executive directors and practitioners (48.1%, 53.3%) perceived their organizations as applying research information 'somewhat well' (see Figures 1 and 2). Executive directors and practitioners were fairly similar in their perceptions of the barriers impeding the application of research information in clinical practice. One-half of the respondents from both groups acknowledged that organizational change is difficult to accomplish, with almost as many feeling unsure about how to make the link between research and practice. One-third of respondents in both groups perceived the generalizability and often conflicting nature of research information and the lack of specific implementation assistance as additional barriers.

Lastly, we considered whether there were any regional differences in how practitioners and executive directors rated the four research utilization constructs across the province. Among practitioners, there were no regional differences in how well they perceived their organizations could *access *research based knowledge, χ^2 ^(24, *N *= 437) = 0.20, *p *> .05; no differences in how well they perceived their organizations could *assess *χ^2 ^(24, *N *= 431) = 0.27, *p *> .05; *adapt *χ^2 ^(24, *N *= 434) = 0.60, *p *> .05, or *apply *χ^2 ^(24, *N *= 425) = 0.89, *p *> .05 research based knowledge. Among executive directors, there were again no regional differences in their perceptions of *access *χ^2 ^(24, *N *= 52) = 0.42, *p *> .05, *assess *χ^2 ^(24, *N *= 51) = 0.51, *p *> .05, *adapt *χ^2^(24, *N *= 51) = 0.92, *p *> .05, or *apply *χ^2^(24, *N *= 52) = 0.34, *p *> .05 research based knowledge

## Discussion

The study surveyed executive directors and practitioners in 80 community-based CMH service provider organizations across Ontario regarding their research utilization practices. There was general agreement among executive directors and practitioners regarding the capacity of their organizations to use – access, assess, adapt, and apply – research evidence. Overall, both groups rated their organizations as using research information 'somewhat well' and both rated their capacity to access and apply more poorly than their ability to assess and adapt. These findings provide a useful benchmark from which changes in reported research utilization in the Ontario CMH sector can be tracked over time, perhaps as a function of EBP training and implementation initiatives.

There is potential to improve access to research evidence, and this should be addressed as it relates to the eventual feasibility of implementing evidence-based practices. With fewer than 40% of organizations connected to the evidence base via academic web-based libraries, there is a significant and actionable need to address this research-practice access gap. Government, service organizations, academic health centres, and universities can make important contributions by partnering to link practitioners to the evidence base within their communities, thereby building linkages among practitioners and scientists at the same time. Practitioners can develop opportunities to network and share practice-based and evidence-based knowledge. The results of this study are consistent with those of Rutledge and Donaldson [49], who found that regional networking combined with tiered continuing education in research can enhance organizational innovation adoption potential by changing organizational communication among nurses.

This study is the first to examine the research utilization practices as reported by the children's mental health workforce. Results highlight a need to improve system and practitioner capacities for research utilization in order to build a base for the implementation of evidence-based practices in the children's mental health sector. Service provider organizations have an important role to play in promoting continuing professional development to enhance practice and evidence-based knowledge and skill.

Fewer than half the executive directors and practitioners perceived their organization as one that could effectively modify research information to meet the needs of their clients and/or programs 'somewhat well.' This supports findings on the complexity of implementing scientific evidence into practice [50]. Since the evidence base is inherently subjective and ever-changing, it is critically important to describe the decision-making processes involved in the interpretation and application of evidence. Very few executive directors and practitioners – less than 10 percent, perceive their organizations as doing 'very well' in the adaptation and application of research evidence in practice.

Barriers to research utilization were similar to those identified in the health literature. Time, money, staff, access to the evidence base, conflicting priorities, organizational commitment, availability and quality of research in this field, formats and venues for knowledge exchange were all identified as barriers in our research with CMH practitioners and executive directors. Lack of physical access to research, including limited access to libraries, has been reported by both policy makers and practitioners as a major barrier to their use of research [51-53]. Many of the barriers practitioners report in using research are related to the organizational contexts in which they work [54]. Among these are lack of time [51,55,56], limited budgets [56] and service user expectations or preferences [57].

We found no evidence of regional differences with respect to how practitioners and executive directors think about their capacity to access, assess, adapt, and apply research based knowledge. However, a recent study on the knowledge translation needs of CMH service providers serving rural areas has shown that while they acknowledge the importance of research evidence and its translation to practice for improving their practice and knowledge, they do not feel ready to discuss the implementation of best practices research. Rather, they expressed that their communities were desperately in need of information about children's mental health services, supports, and resources available within (and beyond) their communities [44].

We submit that the most effective way to develop local practice capacity in these areas is to develop learning communities, or communities of practice [58]. By coming together, practitioners can pool their knowledge and skills to interpret and apply research evidence as a collective. Adaptation of research evidence also requires some element of 'reinvention' in EBP implementation. As such, practitioners must come to understand the 'active ingredients' in specific EBPs so they may be modified without negatively impacting on the expected outcomes. This is an area of science that requires further investigation [59].

The findings must be weighed against the limitations of the practitioner sample size, and the extent to which the findings are generalizable to other jurisdictions. We are fairly confident that the 80 organizations included in this study are representative of community based mental health services for children and youth in Ontario. It cannot be said, however, that the practitioners who responded to the survey are representative of Ontario's CMH practitioners because of the limited response rate. With this in mind, it is noteworthy that the practitioner findings are very consistent with those reported by others in the literature, particularly with respect to the identification of barriers to research utilization.

## Conclusion

Connecting children's mental health service providers to the evidence-based is greatly needed and actionable through the development of partnerships with academic institutions.

The state of research utilization among children's mental health leaders and practitioners shows room for improvement and provides a useful benchmark from which changes in research utilization can be tracked over time.

The need to develop capacity for adaptation and application of research evidence can be accomplished through the development of local or regional communities of practice that can leverage practitioner knowledge and create receptivity for evidence-based practices in this sector.

## Competing interests

The author(s) declare that they have no competing interests.

## Authors' contributions

MB and KMB designed the study. MB developed the survey, for which KMB and ES provided feedback. MB conducted the analysis and drafted the manuscript. ES and KMB conducted the interviews for the larger study. KB and DF contributed conceptually to the literature review of the larger study, and provided literature they had reviewed for a Robert Wood Johnson grant in the US. HBF contributed to the conceptualization of the study and helped to broker the study with decision maker partners. All authors read, made comments and edits, and approved of the final manuscript.
